# Performance of chlorophyll *a* fluorescence parameters in *Lemna minor* under heavy metal stress induced by various concentration of copper

**DOI:** 10.1038/s41598-022-14985-2

**Published:** 2022-06-23

**Authors:** Hanwant Singh, Deepak Kumar, Vineet Soni

**Affiliations:** Plant Bioenergetics and Biotechnology Laboratory, Department of Botany, Mohanlal Sukhadia University, Udaipur, Rajasthan 313001 India

**Keywords:** Biological techniques, Physiology, Environmental sciences

## Abstract

The objective of the present investigation was to understand the efficacy of chlorophyll fluorescence analysis and to identify the specific photosynthetic parameters for early and rapid detection of Cu-induced HM-stress in plants. Aquatic angiosperm *Lemna minor* was exposed to various concentrations (0–40 µM) of Cu. We observed that the F_V_/F_O_ (Efficiency of the water-splitting complex on the donor side of PSII), quantum yield for electron transport, and quantum yield of primary photochemistry were decreased however, dissipated quantum yield was increased with Cu concentration. ABS/CS_M_, TR_O_/CS_M_, ET_O_/CS_M_ and maximum quantum yield were displayed the dose–response relationship under Cu stress. Performance indexes were increased initially due to the beneficial effects of Cu at lower concentration while decreased significantly (*p* ≤ 0.05) at highest concentration of Cu. The outcomes of the present research revealed that the ChlF analysis is very sensitive tool that can be used to determine the toxicity of heavy metals in plants.

## Introduction

In nature, plants are continually exposed to abiotic and biotic stresses. Heavy metals (HMs) like Hg, Cu, Pb, Zn, Ni, Co, Mn, As, etc. have been accumulating in soils for a long time due to anthropogenic activity such as the use of chemical fertiliser, sewage, industrial and smelting wastes^1^. HMs are non-biodegradable elements that cannot be eliminated from the environment through natural processes. Some of them are said to be immobile, that unable to move from the site where they have accumulated, others are referred to as mobile because they can be taken up by the root system of plants via diffusion, endocytosis, or metal transporters^2–13^. However, some metals, such as zinc, copper (Cu), nickel, etc. are important micronutrients that must be absorbed in small amounts as cofactors for several enzymes. Besides these, some HMs found in pesticides such as Cd, Pb, Hg etc. have no beneficial properties and become harmful when their concentration exceeds a threshold limit^14–18^. These HMs may or may not be essential for the proper growth of plants but accumulate in the plants (as a natural accumulator) from soil and water^19–21^. Metal accumulation rates and plant tolerance vary from species to species^22^. Some of the HMs become more toxic than others causing chlorosis, stunted growth, root browning, and mortality are some of the apparent indicators of HM toxicity in plants^23, 24^.

Cu (Cu) is an important element in plants that serves several functions at the physiological and molecular levels. However, the excessive levels of it might constitute a risk to the survival of plants. Cu is a key component of plastocyanin and cytochrome oxidase that are essential for photosynthesis and respiration which have a crucial function in plant carbon assimilation and ATP generation^25, 26^ Cu-stressed plants exhibit a variety of visible symptoms, including chlorosis, stunted development, ion leakage and reduced root growth^27^. Excessive levels of Cu in plants can lead to oxidative stress that causes severe damage to membranes and macromolecules, as well as having a negative impact on many metabolic pathways^28^. Neelima and Reddy investigated the effects of Cu in *Solanum melongena* seeds and revealed that excess Cu reduces germination, seedling length, and root number^29^. All these consequences are extremely harmful to the plant.

Chlorophyll *a* fluorescence (ChlF) is a commonly used method to detect plant stress conditions in plant research, frequently in association with other morphological, chemical, and physiological variables^30–40^. Chl *a* fluorescence (ChlF) is the natural phenomenon describing the dissipation and heat radiation or re-emission of the portion of absorbed energy which is not utilised to drive photosynthesis^23, 41–46^. ChlF measurement provides information about changes in photosynthetic efficiency and heat dissipation^47, 48^. It is an incredibly simple, non-invasive, extremely sensitive, rapid, and accurate method and providing a quantitative assessment of oxygenic photosynthesis^37^. Plants exposed to HM ions disrupt photosynthesis as a result of a single or cumulative event of HM interaction with protein which increase the rate of ROS generation and which replaces essential kations in protein active centers^28^.

Some HM ions, for example, Cu, Hg, Cd, Zn, or Ni can replace the core Mg ion in chlorophyll molecules, resulting in chlorophyll-metal complexes and a reduction in PSII quantum efficiency^49–51^. Apart from evaluating specific parameters, of which the F_V_/F_O_ and F_V_/F_M_ are the most well-known and extensively utilised, the interpretation of double normalised curves using the JIP test is becoming increasingly popular in environmental research practices^42^. Plots are formed using data collected at a high sampling rate within a second of the dark-adapted leaf being exposed to light, as the independent variable on a logarithmic timeline. On such plots, inflection points (J-I-P) are noticed when the recorded fluorescence increases which provide the foundation for inferences regarding the photosynthetic apparatus' structure and function. the O-J-I-P transient is prime source of observed variations in the efficiency of the chlorophyll antenna in capturing light energy and transfer to plastoquinone Q_A_ (the electron acceptor) is the only limitation of photochemical conversion in PSII^52, 53^. Even though, ChlF there are years of in-depth expertise, valid interpretations of ChlF data still require more research^54^. ChlF measurement has become a simple, effective, and dependable technique for outdoor environmental research to improve knowledge and current technology^42, 43, 45, 55–59^.

HM, pollution is becoming more prevalent in the environment, demanding rapid and effective solutions for metal remediation. The use of metal-accumulating plants for remediation has recently given rise to a new technology known as phytoremediation^60^. An ideal hyperaccumulator plant species must meet two requirements for this technology to be viable are HM tolerance and accumulation. Consequently, a better knowledge of the metal tolerance mechanism(s) is critical for the development of effective phytoremediation techniques^61^. The chlorophyll *a* fluorescence has long been used to measure the effects of environmental stress on plants, because they provide a quick approach to determine injury in the absence of visual signs^62, 63^. Therefore, the ability to identify the toxic effects before any morphological symptoms can be seen makes phytoremediation an extremely effective method for identifying metal-tolerant plants.

Duckweeds have high potential to grow under HM stress because of their potential to bioremeidte HMs through either by rhizofilteration or phytotransformation. Therefore, besides use in bioremediations, duckweeds serve a rich source of essential HMs such Cu and Zn for improving feed efficiency of animals^64^.

Chlorophyll (Chl) *a* fluorescence signals have become one of the potent indicators for early detection of HMs in soil and aquatic bodies^25, 65, 66^. In the present study, we used the chlorophyll (Chl) *a* fluorescence transient to investigate the effects of HM stress induced by various concentration of Cu in *L. minor* plants grown in a nutrient medium.

## Materials and methods

### Plant material and growth condition

*L. minor* plants were collected from the region of Ayad river located at Udaipur, India (24° 35′ 14.97′′ N, 73° 42′ 38.75′′ E) (As per the Biological Diversity act, 2002 of National Biodiversity Authority of India, the Indian researchers neither require prior approval nor need to give prior intimation to SBB for obtaining biological resource for conducting research^67^). The plant was identified by Dr. Vineet Soni based on the morphological characteristics (oval shaped fronds, 2–5 fronds remained together, presence of three nerves in each frond and cylindrical root sheath with two lateral wings). The collected fronds (stock culture) were maintained in plastic (PVC) aquariums in Jacob culture media as per the OECD guideline of 2002^68^. The stock culture and Cu treated plants were kept in controlled conditions at 150–230 µmol/m^2^/s (PAR) by using white fluorescent light, 14:10 h light: dark cycle, and 25/20 °C day/night temperature. This medium consisted of the following: Stock solution (A): Ca (NO_3_)_2_, 60.0 g/L, Stock solution (B): MgSO4·7H_2_O, 102.0 g/L; KNO_3_, 100.0 g/L; KH_2_PO_4_, 14.0 g/L, Stock solution (C): H_3_BO_4_, 0.300 g/L; MnCl_2_·4H_2_O, 0.3145 g/L; ZnSO_4_·7H_2_O, 0.0356 g/L; Na_2_MoO_4_·2H_2_O, 0.0118 g/L, Stock solution (D): CuSO_4_·5H_2_O, 0.0125 g/L; FeEDTA (Ethylenediaminetetraacetate acid), 1.8520 g/L. Stock solutions were kept in a refrigerator and growth media prepared by adding 10 mL of each stock to 1 L of distilled water and then adjusting the pH 6.0 using NaOH or HCl^69^.

### Cu exposure

For the ChlF experiment ~ 30 two or three-fronded, healthy plants (300 mg) were taken from stock culture and transferred to glass bottle containing 250 mL of growth medium without EDTA and exposed to various concentrations of CuSO_4_·5 H_2_O (Sigma Aldrich, C8027, ≥ 98%) (0, 10, 20, 30, and 40 μM) for 24 h. The metal exposure experiments were performed according to procedure described by Teisseire and Guy using EDTA free growth medium since it is a chelating agent and alter the metal adsorption process in plants (can increase the bioavailability of metal)^70^. Control plants were grown under both EDTA and Cu free growth medium. The experiment glass bottles were placed in a controlled environment as described above.

### Chlorophyll a fluorescence transient

ChlF was measured using a plant efficiency analyser (Handy PEA fluorimeter, Hansatech instruments Ltd. England). Before measurement fronds were dark-adapted for 50–60 min at 26 °C. Thereafter, ChlF signals were analysed with the Biolyzer v.3.0.6 software (developed by Laboratory of Bioenergetics, University of Geneva, Switzerland). The experiments were done in six replicates and repeated three times to ensure the results. JIP-test method has been developed by which several selected phenomenological and biophysical parameters quantifying the PSII and PSI behaviors are calculated. Several parameters can be derived from the polyphasic ChlF rise (OJIP curve) that provide information about photosynthetic fluxes^41, 71–73^. Abbreviations, formulas, and definitions of the JIP-test parameters used in the current study are presented in Table 1.

**Table 1:** Principal component analysis of chlorophyll *a* flourescence parameters of *L. minor* under various concentration of Cu.

Parameters	Coefficients of PC1	Coefficients of PC2
Tf(max)	− 0.14954	− 0.37572
Area	0.22914	− 0.19298
Fo	0.16258	0.39279
Fv	0.24298	0.11948
Fm	− 0.251	− 0.0207
Vj	0.24927	0.02706
Fv/Fo	− 0.24935	0.04374
Vi	− 0.21397	0.20491
N	− 0.24055	− 0.05733
ABS/RC	− 0.23608	0.16507
TRo/RC	− 0.20833	0.27505
ETo/RC	0.16819	0.38453
DIo/RC	− 0.24915	0.05531
ABS/CSo	0.13162	0.44138
TRo/CSo	0.23242	0.19605
ETo/CSo	0.2498	0.05198
DIo/CSo	− 0.24981	0.05349
PI(abs)	0.22676	− 0.22471
PI(csm)	0.23513	− 0.18388

### Principal component analysis (PCA), grid correlation matrix and heat map

The relations between the selected JIP-test parameters were tested by Principal Component Analysis. ChlF parameter was selected for the PCA analysis to classify the variables that show the maximal fluctuations. Dimension 2 (PC 2) described the maximum of the variability which accounted for 79.15% and dimension 1 (PC 1) accounted for 18.17%, respectively. The positive and negative correlation between the parameters also shows the variation of the parameters in the respective principal components (dimensions) (Table 2). The correlation between all ChlF parameters investigated in this paper were analysed through grid correlation matrix by using Python software which expressed between + 1 and − 1 with colour code. The calculated JIP parameters were also presented by the heat map, through normalizing them between 1 and 100 by using a color code green to red.

**Table 2:** Abbreviations, formulas, and definitions of the JIP-test parameters.

**Basic parameters calculated from the extracted data**	
F_O_ ≅ F_50µs_ or ≅ F_20µs_	Fluorescence when all PSII RCs are open (≅ to the minimal reliable recorded fluorescence)^81^
T_FM_ = tF_MAX_, t for F_M_	Time (in ms) to reach maximal fluorescence F_m_^81, 110^
F_M_ (= F_P_)	Maximal fluorescence, when all PSII RCs are closed (= F_P_ when the actinic light intensity is above 500 µmol(photon) m^−2^ s and provided that all RCs are active as Q_A_-reducing)^81, 110^
F_V_ ≡ F_M_ − F_O_	Maximal variable fluorescence^81^
S_M_ ≡ Area/(F_M _− F_O_) = Area/F_V_	Normalised Area to F_m_^81^
N = S_M_ × (M_O_/V_J_)	Turnover number (expresses how many times Q_A_ is reduced in the time interval from 0 to tF_M_)^81^
V_J_ = (F_J_ − F_O)_/(F_M_ − F_O_)	Relative variable fluorescence at t = 2 ms^81^
V_I_ = (F_I_ − F_o_)/(F_M_ − F_o_)	Relative variable fluorescence at t = 30 ms^81^
**Biophysical parameters derived from the basic parameters**	
Deexcitation rate constants of PSII antenna	
k_N_ = (ABS) × k_F_ × (1/F_M_)	Nonphotochemical deexcitation rate constant (ABS: absorption flux—see below; k_F_: rate constant for fluorescence emission)^42, 81, 110^
k_P_ = (ABS) × kF × (1/F_O_ − 1/F_M_) = k_N_ × (F_V_/F_O_)	Photochemical deexcitation rate constant^81, 110^
Specific energy fluxes (per RC: QA-reducing PSII reaction centre), in ms^−1^	
ABS/RC = M_O_ × (1/V_J_) × (1/φP_o_)	Absorption flux (exciting PSII antenna Chl a molecules) per RC (also used as a unit-less measure of PSII apparent antenna size)^74, 81, 110^
TR_O_/RC = M_O_ × (1/V_J_)	Trapped energy flux (leading to Q_A_ reduction), per RC^81^
ET_O_/RC = M_O_ × (1/V_J_) × (1 − V_J_)	Electron transport flux (further than Q_A_^−^), per RC^81^
DI_o_/RC = ABS/RC − TR_o_/RC	Dissipated energy flux per RC (at t = 0)^81^
Phenomenological energy fluxes (per CS: QA-reducing PSII cross section), in ms^−1^	
TR_O_/CS_M_ = (F_v_/F_M_) (ABS/CS_M_)	Trapped energy flux (leading to Q_A_ reduction) per RC^81^
ET_O_/CS_M_ = (F_v_/F_M_) (1 − V_J_) (ABS/CS_M_)	Electron transport flux (further than Q_A_^−^) per RC^81^
DI_O_/CS_M_ = (ABS/CS_O_) − (TR_O_/CS_m_)	Total energy dissipated per reaction center (RC)^81^
ABS/CS_M_ = ≈ *F*o	Absorbed photon flux per excited PSII cross section at time zero^81^
Quantum yields and efficiencies	
φ_Po_ ≡ TR_0_/ABS = [1 − (F_O_/F_M_)]	Maximum quantum yield for primary photochemistry^81^
φ_Eo_ ≡ ET_0_/ABS = [1 − (F_O_/F_M_)] × (1 − V_J_)	Quantum yield for electron transport (ET)^74^
ψE_o_ ≡ ET_0_/TR_0_ = (1 − V_J_)	Efficiency/probability that an electron moves further than Q_A_^−^^74^
ϕD_o_ = F_o_/F_m_	Quantum yield (at t = 0) of energy dissipation^74^
Performance indexes	
${PI}_{ABS}=\frac{1-({F}_{O}/{F}_{m})}{{M}_{O}/{V}_{j}}\times \frac{{F}_{m}/{F}_{o}}{{F}_{O}}\times \frac{1-{V}_{j}}{{V}_{j}}$	Performance index for energy conservation from photons absorbed by PSII until the reduction of intersystem electron acceptors^74, 81, 110^
${PI}_{CS}=\frac{ABS}{CS} \times \frac{1-({F}_{O}/{F}_{m})}{{M}_{O}/{V}_{j}}\times \frac{{F}_{m}/{F}_{o}}{{F}_{O}}\times \frac{1-{V}_{j}}{{V}_{j}}$	Performance index on cross section basis^74, 81, 110^

### Statistical analysis

Statistical analysis was performed using analysis of variance (ANOVA), followed by a Tukey HSD test (*p* = 0.05) using XLSTAT 2020. Only significant values (*p* ≤ 0.05) of measurements are presented in figures. The heat map was prepared by normalizing the values and bringing them all to a range between 1 and 100 to provide an unbiased color code. Three color code combination of red for high (100%), yellow for medium (50%), and green for the lowest value (1%) was used to represent the heat map. The MS excel and CorelDraw software were used for calculation and designing of the heatmap. In addition, a principal component analysis (PCA) was conducted by eigenvalue decomposition of a data correlation matrix using OriginPro 2016. PCA was applied to find the patterns of the fluorescence parameter and variations in the experimental data. The 48-h lethal dose (LD_50_ and LD_90_) was determine by Probit Analysis using SPSS (22.0). Comparision of mortility ratios between experimental and control groups in the deferent concentrations was performed with Chi-square testing.

## Results

Cu stress significantly altered the growth and productivity of *L. minor* through the modulation of the photosynthetic process. In the present studies, impacts of Cu-induced HM stress on ChlF kinetics, specific energy fluxes, phenomenological energy fluxes, and performance indexes were studied in *L. minor*.

### ChlF rise

**Figure 1:**
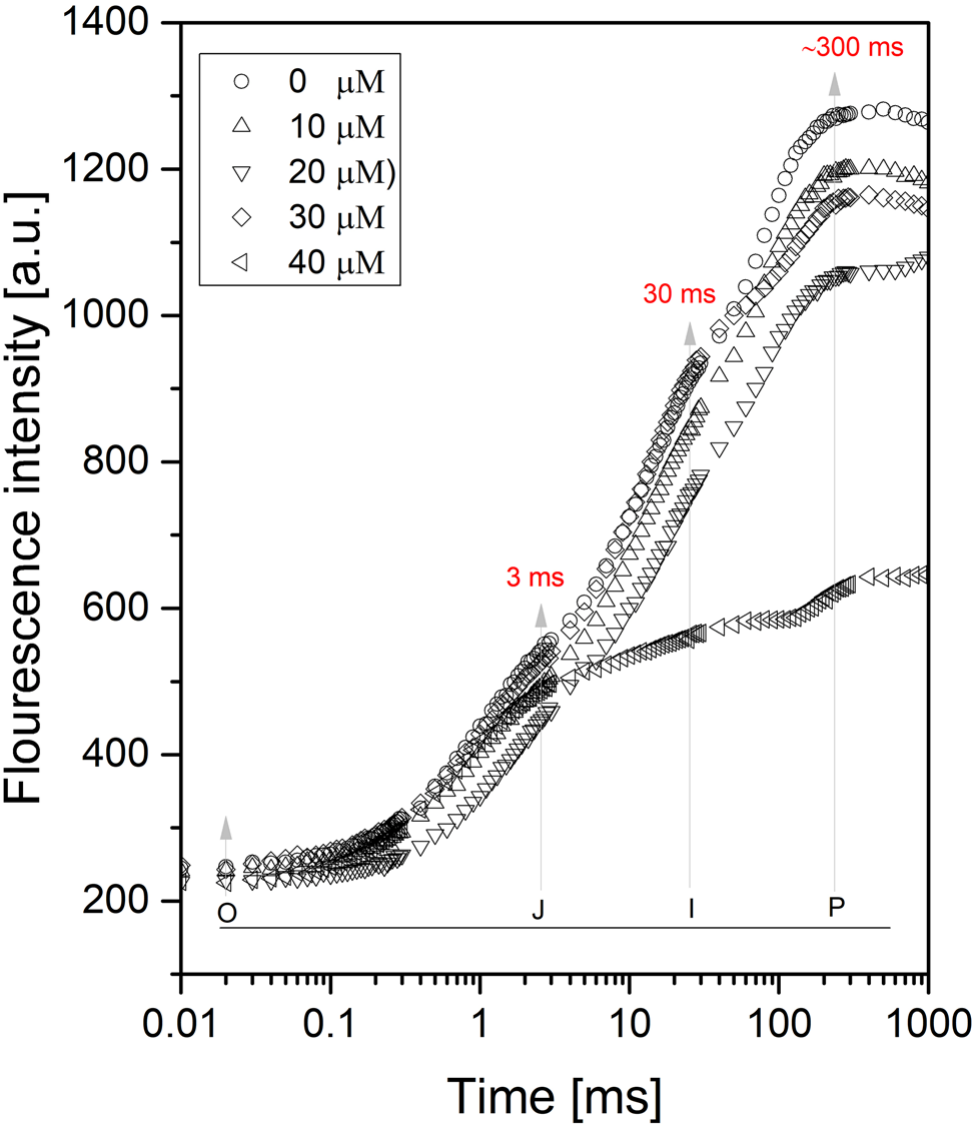
ChlF rises in *L. minor* plants exposed 24 h. to different concentrations of CuSO_4_ (0.0 µM to 40.0 µM) and O, J, I, and P indicate PSII rapid fluorescence transients.

ChlF rise of *L. minor* was measured after 24 h of Cu treatment and a typical OJIP induction curve was displayed when plotted on the logarithm time scale (Fig. 1). With increasing the Cu concentration, the fluorescence yield at various intermediary steps, such as J, I, and P was reduced. In control plants, two intermediate peaks F_J_ (chlorophyll fluorescence at 2 ms) and F_I_ (chlorophyll fluorescence at 300 ms) were formed between F_O_ and F_M_, ChlF increased continuously from initial (F_O_) to maximal (F_M_) fluorescence intensity in *L. minor* growing under control conditions. HM induced reduction in PSII photochemistry and electron transport activity were severe at the highest concentration of Cu.

### Biophysical parameters derived by the ‘JIP-test’ equations’

#### F_O_ and F_M_

**Figure 2:**
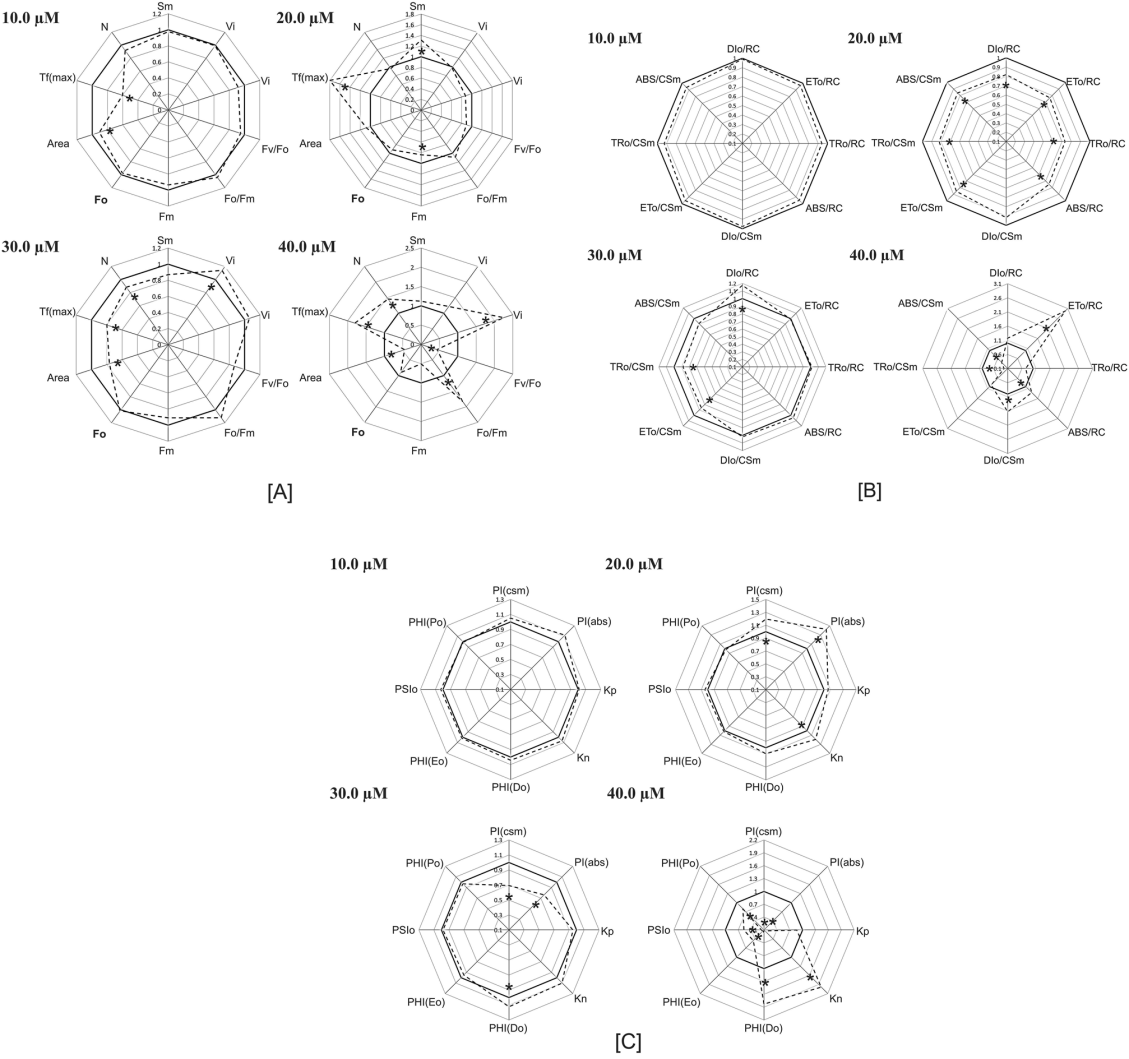
Radar plots (**A–C**) showing various technical fluorescence parameters. Each line represents the average of 6 measurements per treatment. Asterisk denote the significance at *p* ≤ 0.05 level.

The minimal fluorescence intensity (*F*_O_) and the maximum fluorescence intensity (*F*_M_) both are decreased with increasing the Cu concentration (Fig. 2A). Fluorescence intensity recorded at 50 µs is denoted as *F*_O_ and at this time the all primary quinone acceptor (Q_A_) is in the open (oxidized) state.

**Figure 3:**
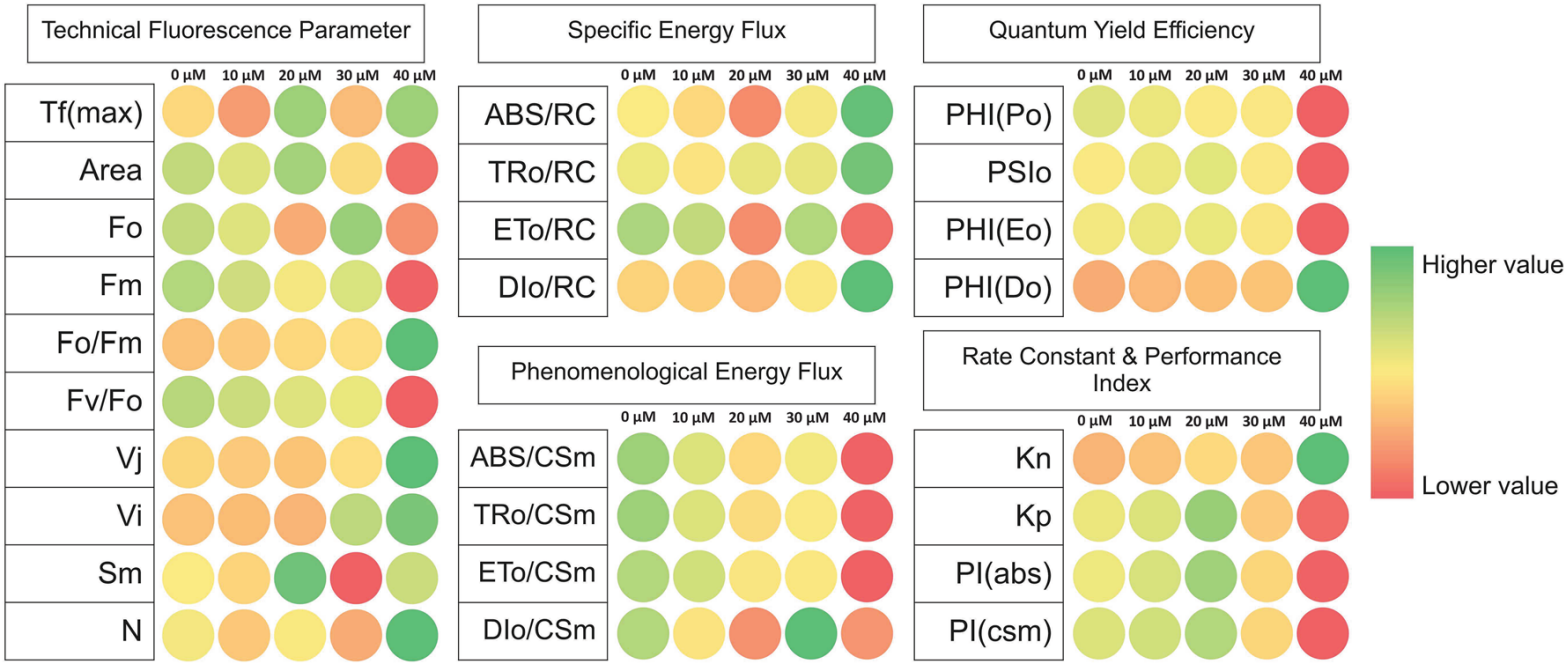
Heat map represents relative variability of several photosynthesis-related parameters, obtained after using the JIP test for *L. minor* under Cu stress. Data are for different concentrations (0.0 µM to 40.0 µM), obtained after 24 h red is for lower value (1%), yellow for medium (50%), and green for the highest values (100%) All the data obtained were first normalized to bring the value of the parameters in the range of 1–100 to provide an unbiased colour code.

The maximum primary yield of photochemistry of PS I1 (*F*_V_/*F*_O_) are related with photosynthetic efficiency of plant and increased value of *F*_V_/*F*_O_ indicates proper functioning of PSII. The *F*_V_/*F*_O_ ratio (ratio between the rate constants of photochemical and nonphotochemical deactivation of excited Chl molecules) for *L. minor* plants decreased gradually at 10 µM (94.86% of control) and 30 µM (89.44% of control) concentration of Cu (Fig. 2A). Further, a significant decline in *F*_V_/*F*_O_ ratio (43.58% of control) was recorded at a high level (40 µM) of Cu exposure as a result of a significant decrease in *F*_V_ (41.00% of control) as shown in Figs. 2A and 3.

The relative variable fluorescence at 2 ms (J step) is denoted as *V*_J_ which is the measure of the fraction of primary quinone electron acceptor of PSII in its reduced state [Q_A_ − /Q_A(total)_]^74^. At the lower level of HM stress, a slight reduction in the value of *V*_J_ was observed but with increasing the metal concentration to a high level the *V*_J_ value was increased to 224.74% of control (Figs. 2A, 3).

Complimentary Area (*S*_M_) is also an important parameter that is directly proportional to the number of reduction and oxidation of one Q_A_^-^ molecule during the fast OJIP transient or number of electrons passing through the electron transport chain^75^. The turnover number (N) is represented as the number of times Q_A_ becomes reduced and re-oxidized another time, till the *F*_M_ (Maximum fluorescence intensity) is reached^76–79^. At severe Cu stress the increased value of turnover number (N) value was recorded (145.40% of control) which was also represented by PSI cyclic electron transport as photoprotection (Fig. 2A). The increased values of *S*_M_ in *L. minor* under Cu exposure (131.40% of control at moderate HM treatment) displayed the reduced electron transport between these photosystems.

#### Quantum yield

The quantum yield of primary photochemistry F_V_/F_M_ (φPo), which reflects the overall photosynthetic potential of active PSII reaction centers, was not affected by Cu-induced HM stress in plants. However, a slight decline in F_V_/F_M_ was recorded at 40 µM Cu (Fig. 2C). A similar trend was observed in ET/ABS (φEo) (Figs. 2C, 3). The lowest values of φEo, approximately half of control, were recorded when *L. minor* was subjected to 40 µM Cu. In contrast, DI/ABS (φDo) remained almost the same until the exposure of 30 µM Cu and thereafter enhanced about two folds of the control level in plants grown in media containing 40 µM Cu (Figs. 2C, 3).

#### Specific energy flux (membrane model)

**Figure 4:**
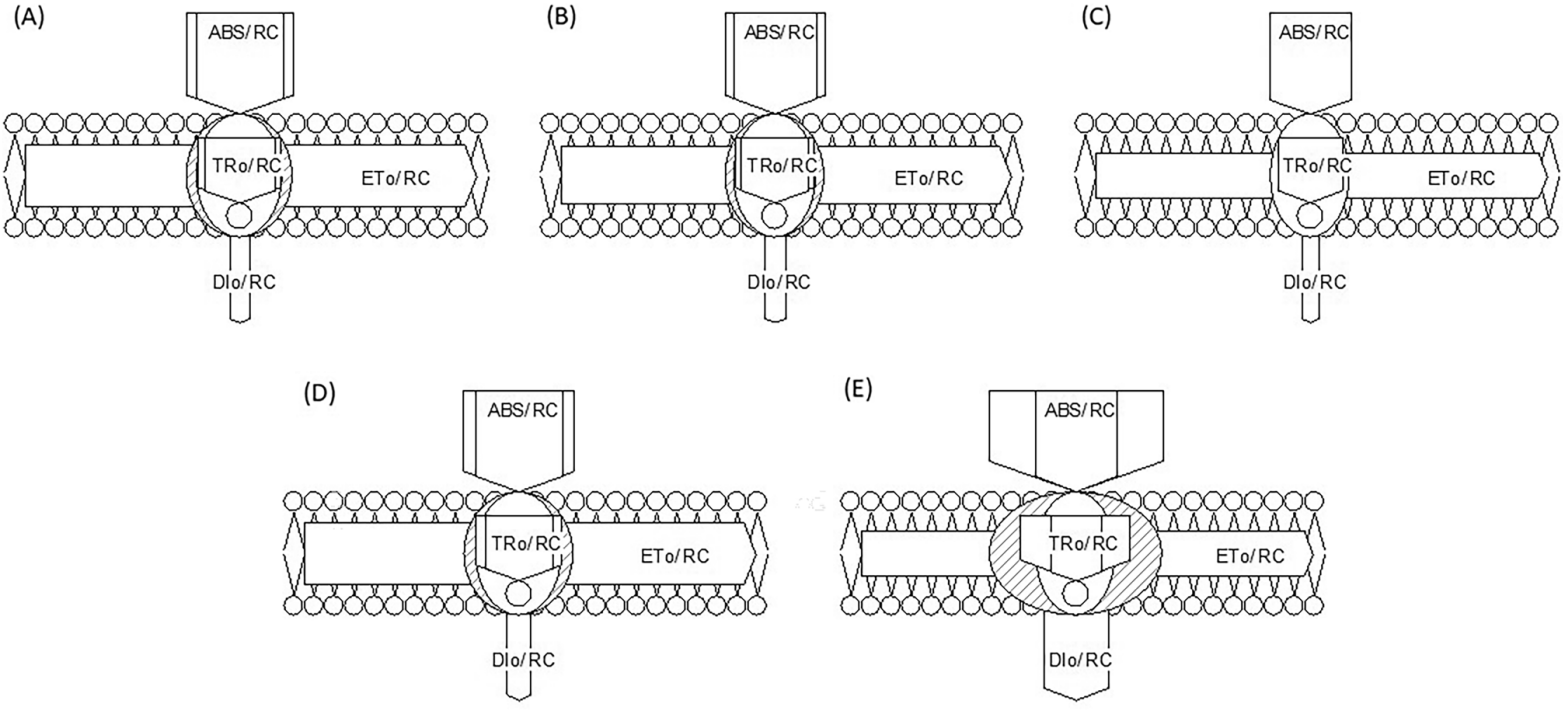
Thylakoid membrane model for specific energy fluxes (per reaction, RC) in *L. minor* fronds when subjected to various concentration of CuSO_4_, (**A**); control, (**B**); 10.0 µM, (**C**); 20.0 µM, (**D**); 30.0 µM and (**E**); 40.0 µM.

The specific energy fluxes such as absorption flux per reaction center (ABS/RC), trapped energy flux per reaction center (TR_O_/RC), electron transport flux per reaction center (ET_O_/RC), and dissipated energy flux per reaction center (DI_O_/RC) were analyzed to determine the photosynthetic performance of active PS II reaction centers of *L. minor* subjected to various concentrations of Cu (Figs. 3, 4). Up to 30 µM Cu, no significant variations in absorption flux per reaction center (ABS/RC) was recorded while at 40 µM, a remarkable enhancement in absorption potential of active reaction centers was recorded (162.83% of control) (Figs. 3, 4). A similar trend in TR_O_/RC was observed as shown in the heatmap (Fig. 3). TR_O_/RC remained almost constant up to 30 µM Cu and thereafter increased with increasing the severity of Cu-induced HM stress (129.68% of control). In contrast, no significant changes in electron transport flux per reaction center (ET_O_/RC) were recorded up to 20 µM Cu concentration while decreased at severe Cu stress (73.58% of control). On the contrary, the DI_O_/RC remained constant up to 30 µM Cu and then increased about three folds with the progression of Cu concentration (297.67% of control) as shown in Fig. 2B. The effects of Cu-induced HM stress on the specific energy fluxes (absorption flux per reaction center, trapped energy flux per reaction center, electron transport flux per reaction center, and dissipated energy flux per reaction center) are presented diagrammatically through thylakoid membrane models (Fig. 4). It is of interest to investigate if HM stress changes the ratio among antenna light-harvesting complex (ABS) and active PSII reaction centers (RC). According to the leaf pipeline model in severe Cu stress, there is more active RC and the higher value of specific energy flux (ABS/RC, TR_O_/RC, and DI_O_/RC) shows the increased ability of RC to the reduction of plastoquinone (Fig. 4).

#### Phenomenological energy flux (leaf model)

**Figure 5:**
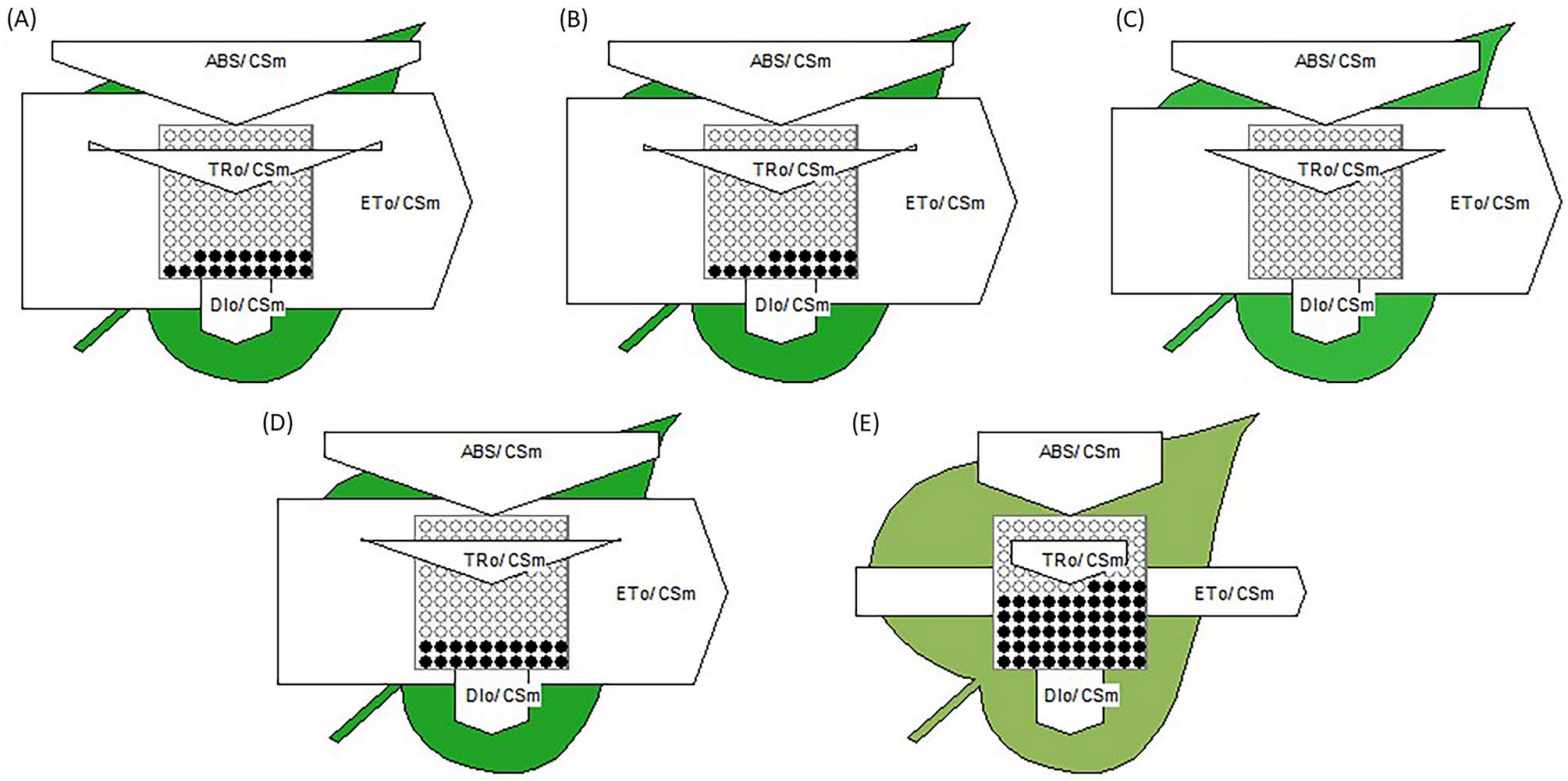
Energy pipeline leaf model of phenomenological fluxes (per cross section, CS) in *L. minor* fronds when subjected to various concentration of Cu, (**A**); control, (**B**); 10.0 µM, (**C**); 20.0 µM, (**D**); 30.0 µM and (**E**); 40.0 µM.

Phenomenological energy fluxes mean absorption flux per cross-section (ABS/CS), trapped energy flux per cross-section (TR_O_/CS_M_), electron transport flux per cross-section (ET/CS), and dissipated energy flux per cross-section (DI_O_/CS_M_) significantly modulated by Cu-induced HM stress in *L. minor*. Absorption flux per cross-section (ABS/CS_M_) did not alter with increasing concentrations of Cu up to 30 µM (Fig. 5). The lowest values of ABS/CS_M_, TR_O_/CS_M_ and ET_O_/CS_M_ were noticed at the highest concentration of Cu (92.09%, 73.34% and 41.61% of control respectively). Absorption potential per cross-section significantly declined when plants were treated with 30 µM Cu for 48 h. TR_O_/CS_M_ reduced remarkably up to 30 µM Cu and thereafter declined up to 50% with increasing concentration of Cu (Fig. 5).

Electron transport efficiency of plants was found tolerant to Cu-induced HM stress. A high concentration of Cu (40 µM) extremely reduced the electron transfer system in thylakoid membranes (Fig. 5). DI_O_/CS_M_ was increased significantly (*p* ≤ 0.05) at 30 µM Cu treatment and after that decreased slightly).

#### K_P_ and K_N_

De-excitation rate constants for nonphotochemical reaction (K_N_) increased under Cu stress and at severe stress conditions K_N_ value approaches 198.46% of the control (Figs. 2C, 3). While de-excitation rate constants for photochemical reaction (K_P_) lowered slightly (86.48% of control) at 40 µM Cu concentration.

#### Performance index

Overall effects of Cu-induced HM stress on various photosynthetic parameters are presented in the form of a radar plot (Fig. 2). To analyze the effects of Cu-induced HM stress on overall photosynthesis performance, PI_ABS_ and PI_CS_ were determined in *L. minor* exposed to various intensities of Cu stress. Cu stress led to a significant effect on the performance index on absorption basis (PI_ABS_) and performance index of PS II and PS I (PI_CS_) in *L. minor*. PI_ABS_ and PI_CS_ continuously increased with increasing concentration of Cu up to 30 µM, and then declined sharply with the progression of Cu-induced HM stress. The lowest performance index on absorption basis (PI_ABS_) and performance index of PS II and PS I (PI_CS_) were recorded in plants cultivated on media containing 40 µM Cu (Figs. 2C, 3).

**Figure 6:**
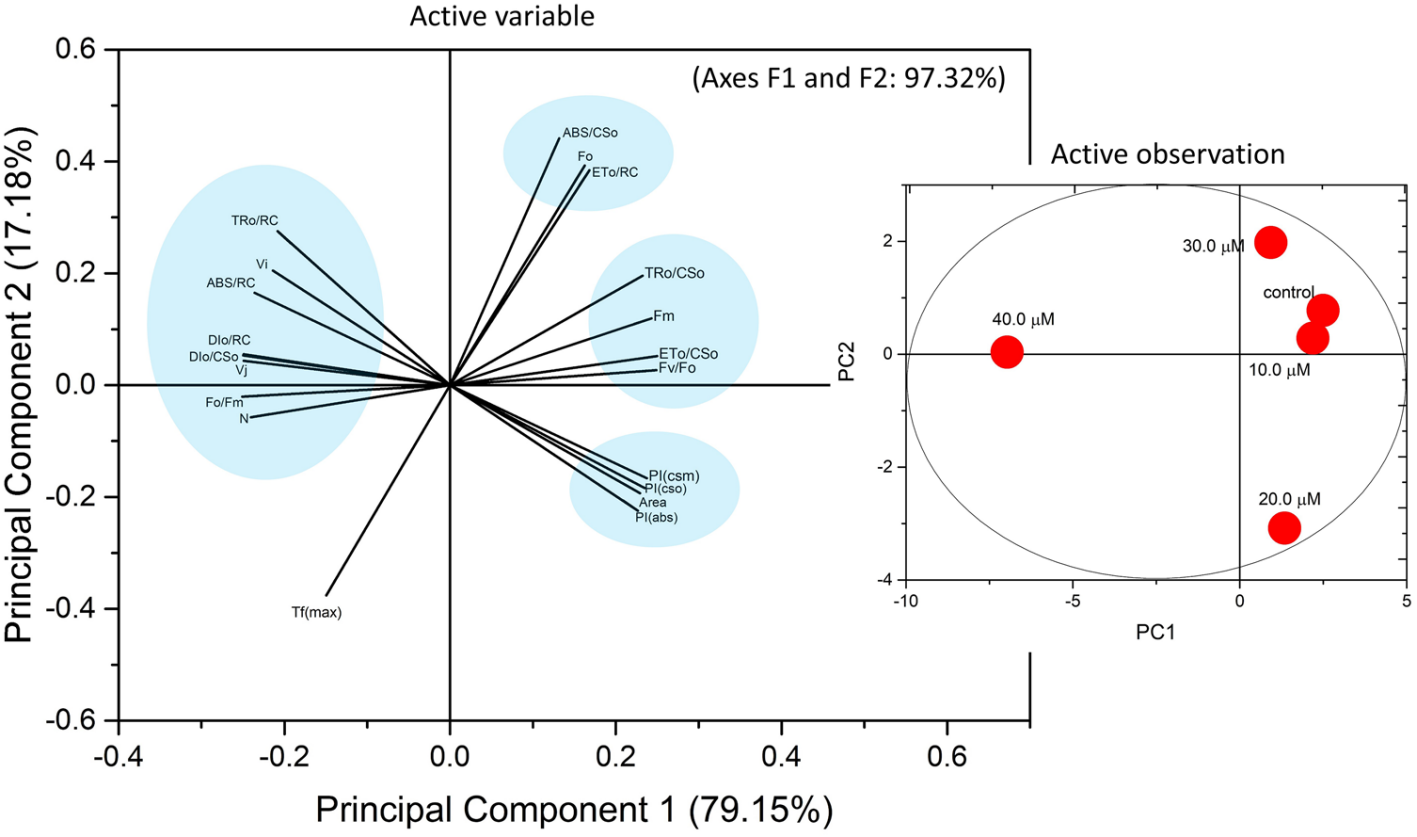
The principal component analysis with four Cu treatment conditions. The PCA is based on the chlorophyll fluorescence data. Arrows represent the Chlorophyll *a* fluorescence parameter on the corresponding dimensions (PC 1 and PC2), where PC 2 expressed most of the variability in the data.

The PCA results displayed that Dim 1 and Dim 2 represented 97.32% of the variation in the ChlF parameter under Cu induced HM stress in *L. minor* (Fig. 6). The loadings for ABS/CS_O_, ET_O_/RC, TR_O_/CS_O_, ET_O_/CS_O_, F_V_/F_M_, PI_CS_ and PI_ABS_ are in quadrate I and IV while, TRo/RC, ABS/RC, DI_O_/RC, DI_O_/CS_O_ and F_O_/F_M_ are accounted in quadrate II and III. All treatments, except 40 µM, are located in quadrate I and IV. The loading arrow of 40 µM is longer than others in all quadrates. Thus, the higher concentration of Cu was significantly affecting the major JIP parameters located in quadrate II and III. The mild Cu stress up to 20 µM was less toxic as compared to severe stress and plants performed better which was described by performance index parameters in quadrate IV (Fig. 6).

In Table 3, values of the lethal dese responsible for 50% mortality (LD_50_) and 90% mortality (LD_90_), calculated by probit analysis with 95% probability level, are given. The LD_50_ and LD_90_ values of Cu was 25.70 µM and 87.80 µM respectively.

**Table 3:** The acute 48–h LD_50_ values of Cu and their confidance limits in *L. minor* according to Logit analysis.

Confidence limits							
	Probability	95% Confidence limits for Cu dose			95% Confidence limits for log(Cu dose)^a^		
		Estimate	Lower bound	Upper bound	Estimate	Lower bound	Upper bound
PROBIT	0.010	2.763	0.422	5.580	0.441	− 0.375	0.747
	0.020	3.588	0.685	6.693	0.555	− 0.165	0.826
	0.030	4.235	0.931	7.516	0.627	− 0.031	0.876
	0.040	4.798	1.172	8.204	0.681	0.069	0.914
	0.050	5.310	1.414	8.812	0.725	0.150	0.945
	0.060	5.789	1.658	9.368	0.763	0.220	0.972
	0.070	6.244	1.906	9.886	0.795	0.280	0.995
	0.080	6.682	2.159	10.376	0.825	0.334	1.016
	0.090	7.107	2.417	10.845	0.852	0.383	1.035
	0.100	7.522	2.682	11.297	0.876	0.429	1.053
	0.150	9.515	4.115	13.413	0.978	0.614	1.128
	0.200	11.469	5.756	15.441	1.060	0.760	1.189
	0.250	13.462	7.637	17.517	1.129	0.883	1.243
	0.300	15.545	9.774	19.759	1.192	0.990	1.296
	0.350	17.762	12.164	22.310	1.249	1.085	1.348
	0.400	20.158	14.767	25.378	1.304	1.169	1.404
	0.450	22.783	17.506	29.257	1.358	1.243	1.466
	0.500	25.700	20.295	34.319	1.410	1.307	1.536
	0.550	28.990	23.102	40.998	1.462	1.364	1.613
	0.600	32.765	25.971	49.837	1.515	1.414	1.698
	0.650	37.184	29.004	61.631	1.570	1.462	1.790
	0.700	42.487	32.338	77.676	1.628	1.510	1.890
	0.750	49.062	36.167	100.261	1.691	1.558	2.001
	0.800	57.588	40.792	133.789	1.760	1.611	2.126
	0.850	69.413	46.768	187.937	1.841	1.670	2.274
	0.900	87.800	55.358	289.186	1.943	1.743	2.461
	0.910	92.928	57.637	321.035	1.968	1.761	2.507
	0.920	98.838	60.211	359.670	1.995	1.780	2.556
	0.930	105.771	63.165	407.596	2.024	1.800	2.610
	0.940	114.091	66.626	468.782	2.057	1.824	2.671
	0.950	124.381	70.792	549.948	2.095	1.850	2.740
	0.960	137.663	76.006	663.573	2.139	1.881	2.822
	0.970	155.951	82.924	836.132	2.193	1.919	2.922
	0.980	184.076	93.072	1137.301	2.265	1.969	3.056
	0.990	239.053	111.575	1848.088	2.378	2.048	3.267

^a^Logarithm base = 10.

## Discussion

Many studies on the plant’s physiological changes under various HM stress have been reported. These studies indicated that plants have developed a series of mechanisms to protect themselves from these adverse environmental threats. ChlF analyses have been shown to detect complex biochemical alteration in photosynthetic apparatus in a vast range of plant species, including both terrestrial and aquatic^80^. The present investigation shows the Cu induced changes in various fluorescence parameters of photosystem II in duckweed *L. minor*.

### ChlF rise

Excess energy enhanced the utilisation capacity of plants in extreme environments, after which photoprotective systems quenched the extra Chl radical, and the extra energy was dissipated as heat^79, 81, 82^. The reduction in ChlF from PSII is being used to quantify these mechanisms, which are jointly referred to as non-photochemical quenching (NPQ)^41, 83, 84^. The J-I and I-P, and the J step still appeared at the severe Cu stress (40 µM), indicating the tolerance capacity of the plant^85^. The O-J is a photochemical phase and J-I-P is a thermal phase, these are two characteristics of OJIP transient and presented three various reduction processes of the electron transport chain^52, 74, 86, 87^. The photochemical phase (O-J) is mainly light-dependent and comprises information regarding antenna size and connection between PSII reaction centers^88, 89^. Further, the reduction in remaining ETC is denoted by the thermal phase (J-I-P) rise^90^.

### Biophysical parameters derived by the ‘JIP-test’ equations’

The values of minimal fluorescence intensity are an important parameter and can provide insight in the irreversible damage of PSII, associated with light-harvesting complex II (LHCII) and hindering the electron transfer on the reduced side of PSII^83, 91^. Because of conformational changes in the D1 protein under Cu stress, which cause changes in the characteristics of PSII electron acceptors, decreasing *F*_M_ under HM stress may be related to less efficient PSII activity^83^.

**Figure 7:**
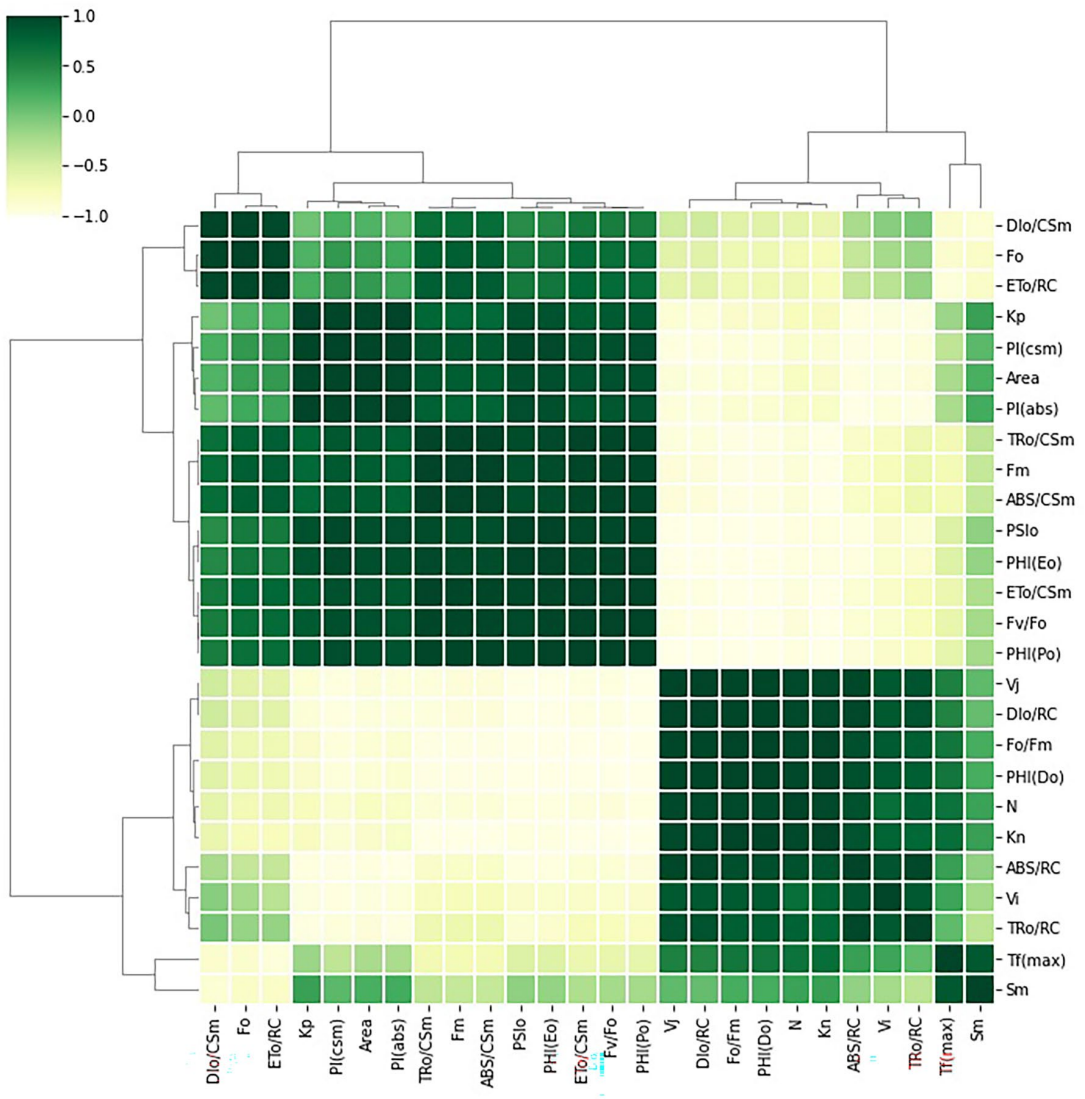
Grid correlation matrix shows the correlation between all calculated chlorophyll *a* fluorescence parameter with color code.

In determining the maximum primary yield of photochemistry, *F*_V_/*F*_O_ is a parameter that accounts for simultaneous variations in *F*_M_ and *F*_O_^45^. The decreased values of *F*_V_/*F*_O_ in fronds under Cu stress show the alteration in the electron transport rate to the primary electron acceptors from PSII and a reduction in the number and size of the reaction center. Martinazzo et al. and Janka et al. also reported the environmental stress-induced decrease in the *F*_V_/*F*_O_ ratio in different plant species^92, 93^. The increased level of relative variable fluorescence under Cu treatment indicates that the electron transfer at the donor side of PSII was affected. The affected *F*_V_/*F*_O_ can be due to the modified unquenchable fluorescence (F_O_) that altered the energy relay from antenna complex to reaction center^94^. According to PCA analysis the quantum yield was positively correlated with the electron transport per cross-section while negatively correlated (Fig. 6) with F_O_/F_M_ located in the opposite direction of the PCA loading plot, which was also confirmed by the Correlation matrix (Fig. 7). Another possibility of reduced maximum primary yield under Cu stress can be the substitution of central atom of chlorophyll molecule, Mg by Cu. This substitution can hinders photosynthetic light-harvesting in the affected chlorophyll molecules^95^.

The “JIP test” of fluorescence transient in photosynthetic organisms, subjected to abiotic stress revealed a marked decrease in φPo^96^. The slight reduction in φP_O_ might be due to a decrease in PSII photochemical efficiency resulting from Cu stress (in most higher plants having usually a value in the range of 0.78–0.84^97^). In the light condition, a reduction in the maximum quantum yield of PSII (φPo) shows that HM stress inhibits the redox reaction following Q_A_ and causes a delay in electron transport between Q_A_^−^ and Q_B·_^90^. These parameters are very important and provide relevant information on electron transport activity at the PSII acceptor sites. The present finding suggested that Cu treatment reduces the electron transport at the PSII acceptor site in *L. minor.*

Energy pipeline models (membrane and leaf model), presented in Figs. 4 and 5, have displayed that various sites in PSII are sensitive to several environmental stresses^98–100^. Based on present results, TR_O_/CS_M_ and ET_O_/CS_M_ decreased with increasing the Cu concentration because active RCs are converted into inactive or closed (dark circle in model) RCs consequently decreasing the trapping efficiency and electron transport from PSII^74, 81, 101^. PCA biplot shown ET_O_/CS_M_, DI_O_/CS_M_ are positively correlated, which is also observed by grid correlation matrix (Figs. 6, 7). The ABS/RC is determined by taking the total amount of photons absorbed by Chl molecules throughout all RCs by the total number of active RCs^58^. The ratio of active/inactive RCs influences it, and as the number of active centers rose, the ABS/RC ratio reduced. TR_O_/RC is the maximum rate at which an exciton is captured by the RC, resulting in a decrease in Q_A_. An increase in this ratio indicates that all the Q_A_ has been reduced^83^. Reduction in ET_O_/RC describes that the re-oxidation of reduced Q_A_ through electron transport in an active RC is decreased because a greater number of the active RC are available, hence it only reflects the activity of active RCs. Figure 4 demonstrates a reduction in per active RC electron transport but an overall increase in electron transport. The ratio of total dissipation of un-trapped excitation energy from all RCs to the number of active RCs is defined as DI_O_/RC. Dissipation arises as heat, fluorescence, and energy transfer to other systems and the ratios of active/inactive RCs also have an impact. Due to the effective utilisation of energy by the active RCs, the ratio of total dissipation to the number of active RCs (DI_O_/RC) is not very impacted^102, 103^.

The F_V_/F_M_ ratio = (F_M_ − F_O_)/F_M_ is an important JIP parameter that represents the conversion efficiency of primary light energy in the PS II reaction center and is used as a stress indicator in a large number of photosynthetic studies^53, 82, 87^. However, since it is dependent on F_O_ and F_M_ fluorescence levels, this quantitative parameter is not usually sensitive enough to detect alteration across samples. Srivastava et al*.* employed the performance index (PI), a novel, more responsive, and significant parameter to measure photosynthesic efficiency under stress^104^. The performance index, PI, is derived using three (or four) components based on reaction center density, trapping efficiency, and electron transport efficiency, in the same way as a Goldman equation^105^. As a result, if any of these components is affected by stress, the effect will be visible in the performance index, which has a higher sensitivity. Performance index (PI_ABS_) is calculated on an energy absorption basis while the performance index on cross-section (PI_CS_) is obtained by multiplying the performance index on absorption basis PI_ABS_, by the phenomenological energy flux, ABS/CS = Fo (or F_M_): and the value of PIabs and P_CS_ significantly lowered in a plant grown under Cu stress (Fig. 2C). PI_ABS_ are decreased due to reduced activity of the RC so the overall activity of the RC is decreased^41, 83, 106^ based on results in this study and statistical models (PCA and Correlation matrix) some of the important JIP parameters such as Phenomenological energy flux (ABS/CS_M_, TR_O_/CS_M_ and ET_O_/CS_M_), maximum quantum yield (φP_O_), Performance index per absorbance (PI_ABS_) and per cross-section (PI_CS_) are displayed the dose–response relationship under Cu stress. Probit analysis is usually used in toxicology to determine the relative toxicity of chemicals to living organisms^107^. Copper LD50 values (Table 3) demonstrated that this molecule can be considered as highly toxic to *L. minor*. Copper phytotoxicity was assessed through the visible symptoms of toxicity and determination of the concentration that results in a 50% reduction in the growth of L. minor (LD 50). According to Teisseire & Guy (2000), CuSO4 at 10 μM was inhibitory for *L. minor* (Teisseire and Guy). However, some plant species tolerate this element at concentrations higher than those used in medium cultures. Our study indicated that, *L. minor* was sensitive to copper for concentrations ≥ 25 µM^108^. This is caused by the different duckweed species used and by the different test conditions, especially concerning the nutrient media as well as by the methods of evaluation (Appenroth et al.^109^).

## Conclusion

In the present study, the efficacy of ChlF kinetics in the detection of Cu-induced HM stress was analysed in *L. minor*. Treatment of lower concentration of Cu (0.0–20.0 µM) had mild negative effect on photosynthesis. As the Cu is an essential micronutrient and plays a vital role in many biochemical processes, hence under moderate metal concentration the *L. minor* performed normally without any deleterious effect. A typical OJIP curve was obtained which shows that the plant efficiently used the solar energy for photosynthesis which is expressed in the term of increased active reaction center and performance index. In contrast, at higher Cu concentration (30.0–40.0 µM), the OJIP curve has been flattened due to a reduction in electron transport towards PSI (P_700_), and a major portion of absorbed energy was dissipated in the form of heat because of an increased number of the inactive reaction center. Conclusively, phenomenological energy flux (ABS/CS_M_, TR_O_/CS_M_ and ET_O_/CS_M_), maximum quantum yield (φP_O_), performance indexes (PI_ABS_ and PI_CS_) are powerful indicators of HM stress in plants and can be used for rapid detection of HM-induced water pollutant.Additionally, the key OJIP parameters screened in this paper could be a good tool for the rapid detaction of primary mode of action of HM on the photosynthetic apparatus in *L. minor*.

## Data Availability

The datasets used and/or analysed during the current study available from the corresponding author on reasonable request.
